# Through the looking glass: how do marked dolphins use mirrors and what does it mean?

**DOI:** 10.1007/s10071-022-01680-y

**Published:** 2022-09-20

**Authors:** A. Loth, O. Güntürkün, L. von Fersen, V. M. Janik

**Affiliations:** 1 Scottish Oceans Institute, School of Biology, University of St. Andrews, Fife, KY16 8LB UK; 2 Biopsychology, Institute of Cognitive Neuroscience, Faculty of Psychology, Ruhr-University Bochum, 44780 Bochum, Germany; 3 Tiergarten Nürnberg, Am Tiergarten 30, 90480 Nuremberg, Germany

**Keywords:** Self-recognition, Theory of mind, Consciousness, Bottlenose dolphin, *Tursiops truncatus*

## Abstract

Mirror-guided self-inspection is seen as a cognitive hallmark purportedly indicating the existence of self-recognition. Only a few species of great apes have been reported to pass a standard mark test for mirror self-recognition in which animals attempt to touch a mark. In addition, evidence for passing the mark test was also reported for Asian elephants, two species of corvids, and a species of cleaner fish. Mirror self-recognition has also been claimed for bottlenose dolphins, using exposure of marked areas to a mirror as evidence. However, what counts as self-directed behaviour to see the mark and what does not has been debated. To avoid this problem, we marked the areas around both eyes of the animals at the same time, one with visible and the other with transparent dye to control for haptic cues. This allowed the animal to see the mark easily and us to investigate what side was exposed to the mirror as an indicator for mark observation. We found that the animals actively chose to inspect their visibly marked side while they did not show an increased interest in a marked conspecific in the pool. These results demonstrate that dolphins use the mirror to inspect their marks and, therefore, likely recognise a distinction between self and others.

## Introduction

Mirror use for self-inspection is considered a cognitive hallmark that has been hailed as an experimental indicator of self-recognition (Mashour and Alkire 2013). The most convincing evidence for successful mirror use in this context comes from experiments in which animals reach for novel marks on their bodies that they cannot see without a mirror, using a mirror to guide their movements. This test has been applied successfully in great apes (Anderson and Gallup 2011), rhesus monkeys (Chang et al. 2015), two species of corvids (Buniyaadi et al. 2020; Prior et al. 2008, but see Soler et al. 2014) and one elephant (Plotnik et al. 2006). However, it cannot be used in animals that either do not have the dexterity to reach for marks or that lack suitable limbs altogether such as fish or cetaceans. In such cases, simplified versions of the test have been used that measure parameters like the approach time to a known mirror and the time spent looking at a mark. Applying such tests has led to a much wider range of taxa being reported to purportedly show mirror self-recognition (Kakrada and Colombo 2022).

Interpretations of mark tests have varied widely in the past, with some researchers arguing self-exploration in front of a mirror was suggestive of self-awareness or even consciousness (Gallup 1983, 1985; Povinelli et al. 1993), whereas others argued it might just demonstrate kinaesthetic matching (Mitchell 1993). Researchers have remained divided with many suggesting that self-exploration in front of a mirror as tested in mark tests serves as evidence for self-recognition (de Veer and van den Bos 1999; de Waal 2019) while a more conservative view is that it only demonstrates a general ability to collate representations (Suddendorf and Butler 2013). While these distinctions are not clear cut, it is likely that the ability to differentiate between self and others emerged gradually (de Waal et al. 2005; Feinberg and Keenan 2005; Kakrada and Colombo 2022; Rochat 2003; Toda and Watanabe 2008), making its study in other species an important step towards understanding the evolution of consciousness (de Waal 2019).

Bottlenose dolphins have highly evolved social and cognitive skills (Connor 2007; Güntürkün 2014; Herman 2006; Janik 2013; Norris and Dohl 1980; Pack 2018; Tyack 1999), and mirror self-recognition has been claimed here as well (Reiss and Marino 2001). Dolphins in zoos often have windows in their environment and the illumination of the tank with a dark surrounding make these good reflectors. Furthermore, dolphins are continuously faced with the reflective water surface when approaching it to breathe (Dibble et al. 2017). Thus, these animals are likely to have a lot more experience with reflections than most other animals tested in these paradigms. Studies on marked bottlenose dolphins have reported that they move their bodies in front of a mirror in a way that allows them to see the marked area and that they do this for longer when a mark is visible rather than transparent (Morrison and Reiss 2018; Reiss and Marino 2001). However, the methods used in these studies have been criticised (Gallup and Anderson 2018, 2020; Güntürkün 2014; Harley 2013; Manger 2013). The main problems have been the absence of suitable controls and the equivocal categorisation of movement behaviour of the animal in front of a mirror as mark-directed, self-directed or social behaviour. A more recent study tried to differentiate such behaviours in more detail (Morrison and Reiss 2018), but it is still unclear how behaviours were determined to be self-directed or not. Thus, further studies on mirror self-recognition in dolphins are needed.

We investigated mirror self-recognition in bottlenose dolphins by use of an adapted mirror-mark test procedure. We marked the animals in two separate locations at the same time, applying a circular mark around each eye, one of which was transparent whereas the other was a visible mark. Thus, we could differentiate between an unspecific interest in mirrors after handling and a specific decision by the animal to inspect the side with the visible mark over the one with the transparent dye because the animal did not have to twist or turn to see the mark. We also investigated whether the interest in the mirror could be explained by an interest in marks on conspecifics more generally.

## Methods

**Fig. 1:**
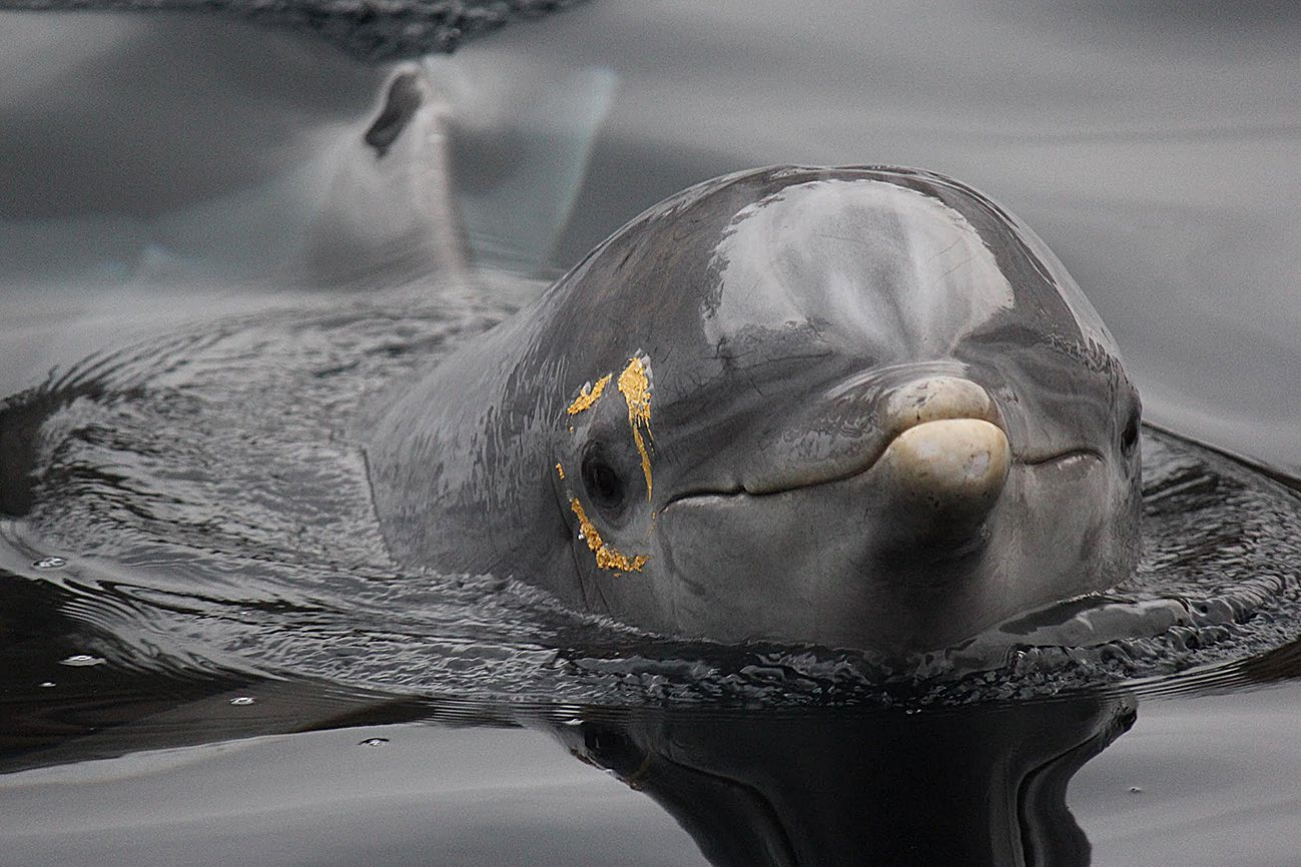
A dolphin marked with yellow dye on its right eye and transparent on its left for a mark test

Four bottlenose dolphins (3 4-year-old males named Kai, Darwin and Diego, and one 26-year-old female named Jenny) were tested at Zoo Duisburg and Tiergarten Nürnberg in Germany, two at each facility. For a mirror-mark session, one animal was temporarily separated from the rest of the group and marked in one of three different conditions: transparent” (both eyes marked with transparent dye), “left” (left eye yellow, right eye transparent) and “right” (right eye yellow, left eye transparent) (Fig. 1). We chose yellow because it provides a high contrast to the prevailing green and blue underwater and because of its shorter wavelength is not absorbed as quickly as red or orange. Dolphins have monochromatic vision (Hanke et al. in press) so that they cannot perceive the actual colour of the mark. The animal was kept separated from conspecifics for 30 min, and the times that it spent looking at a newly installed mirror were measured. Other dolphins could watch the marked individual through the separating nets or fences. All applicable national and institutional guidelines for the care and use of animals were followed. All the procedures performed in studies involving animals were in accordance with the ethical standards of the University of St Andrews (University of St. Andrews Animal Welfare and Ethics Committee).

### Subjects and housing

Data were collected between August and December 2014 (Nürnberg) and between September and December 2015 (Duisburg). All animals were housed in multi-pool-systems in social groups but were temporarily separated by meshed gates for the tests described here. Due to a daily training routine, individuals were fully habituated to short-term separation and the marking procedure. Even though reflections are a daily part of a dolphin’s environment (e.g. surface, windows), none of the animals had previous experience with a mirror or was ever reinforced by a trainer to interact with it. To familiarise them to the presence of the mirror and the cameras as novel objects in the pool, the reflective mirror side was covered with an opaque foil and all equipment was slowly introduced to the pool in sessions varying in duration (between 5 and 25 min). After being completely habituated to the objects in the pool, the animals received a varying number of habituation sessions with the uncovered mirror. Each of these sessions was 30 min in duration and had the same protocol as the later marking sessions with one exception: the animals never received a marking during the medical training parts of these habituation sessions (medical training lasted approximately 5 min at the start of each session). Often contingency checking behaviour (highly repetitive movements in front of the mirror) is used as a criterion to end the habituation and start marking the animal. Since we did not observe contingency checking during habituation sessions, we started with marking sessions as soon as the frequency of mirror interactions decreased (mean habituation time ± SE = 180 ± 45 min).

### Experimental design and setup

**Fig. 2:**
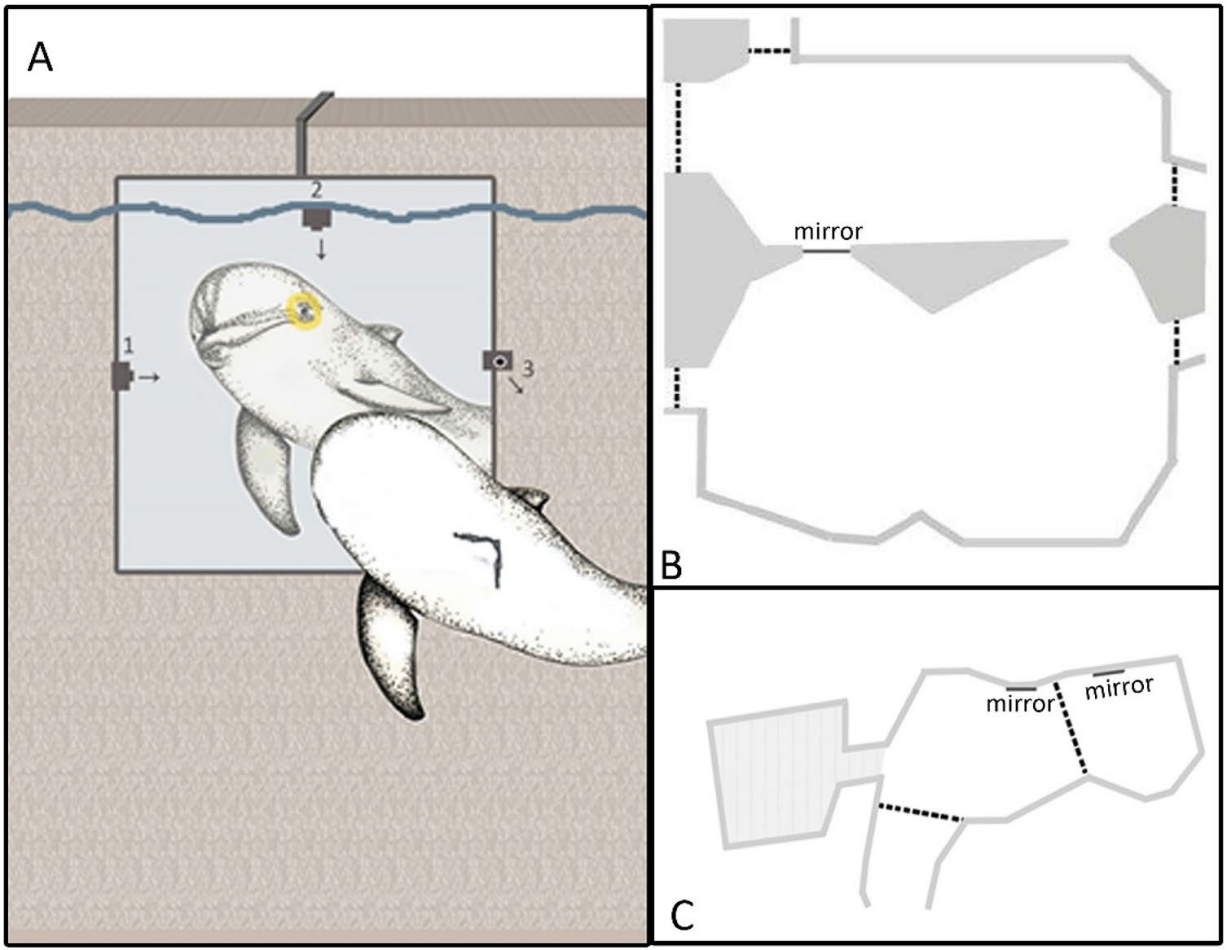
Experimental setup with the acrylic mirror in the pool system. **A** Mirror attached to the pool wall (Duisburg) with under water cameras filming different angles as indicated by the arrows. Grey pool area with very shallow water was accessible but rarely entered. Dashed lines indicate separators such as nets or fences that were closed to separate animals but allowed dolphins to see each other. **B** Test pool in Nürnberg with the mirror integrated into a gate between two pools. **C** Test pools in Duisburg with the mirrors attached to the pool walls

An acrylic mirror (104 cm × 139 cm) was fixed to an opaque PVC board and only presented in the pool during the 30 min of each experimental session (Fig. 2a). In Nürnberg, the two individuals were tested with a mirror integrated into a gate between two pools (Fig. 2b). They could use both pools and interact with the reflective mirror front as well as with the non-reflective backside. In Duisburg, the mirror was attached to a pool wall (Fig. 2c). Dolphins were marked circularly around the eye during general medical training prior to each test session. The trainer always had both the yellow and the transparent dye on separate fingers of each hand, regardless of the marking treatment that was applied in the particular session. In addition, the eye was approached with both fingers at a minimum angle to the animal’s side and only the finger with the correct dye touched the dolphin’s skin minimising the risk that the dolphin could see the marking treatment before looking at its reflection. We always applied dye to both eyes, either all transparent or with colour on one eye or the other. This bilateral marking procedure was used to account for any behavioural reaction that could have been caused by the haptic experience of the marking. The transparent dye contained Vaseline and methylcellulose; for the yellow dye iron oxide and titanium dioxide were added to achieve a high contrast to the dark grey skin without changing the dye’s texture. Both dyes were odourless and water-resistant for up to 45 min. During test sessions, no observer or trainer was present around the pool and no signal or food reward was given to the animals. Experimental treatments (transparent, left, right) were randomised and repeated between 2 and 4 times for each individual (mean ± SE = 3.17 ± 0.83).

### Behavioural coding

Behavioural data were collected with underwater cameras (Gopro Hero3+ and Qumox HD1080P) (Fig. 2a). We measured the occurrence and duration of all exposures of the left or right eye to the mirror (at a maximum distance of 2 m and when the animal was at 90° ± 30°) on the video files using Solomon Coder beta 15.11.19. This included times when the animal was stationary in front of the mirror but also times when it just swam by the mirror at close range and had either the left or right side briefly exposed to the mirror. To test for possible inspections using the water surface as a mirror, we also coded all occurrences when the animal was at less than 1 m of depth and had one eye towards the surface. To calculate the inter-rater reliability, 30% of the videos were randomly picked and re-coded by a second rater who was naïve to the experimental treatment. Calculations showed a high level of agreement between both raters (Spearman’s rho correlation: correlation coefficient = 0.943, *p* ≤ 0.005). In previous studies, behaviour patterns in front of mirrors were often interpreted as self-directed or not. Similarly, rapid approaches to the mirror after marking were interpreted as an intention to self-inspect with the alternative (seeking proximity to another dolphin after an unusual event) often ignored, perhaps because it suggests that self-recognition is absent. We think such behavioural categorisations and interpretation are inherently subjective and they have also been criticised in the literature (Gallup and Anderson 2018; Harley 2013; Manger 2013). We tried to avoid such bias using a measureable response variable (looking time) that is unequivocal.

### Statistical analysis

To assess the influence of the marking treatment on the dolphins’ side orientation in front of the mirror, generalised linear mixed effects models were performed using R version 3.2.2 and the R package lme4 version 1.1-9 (59) with a binomial family, logit link function, and a Poisson error distribution. Within all test sessions, the three males showed a strong preference for a counter-clockwise swimming direction. Therefore, they were generally more likely to expose their right eye to the mirror. We used the orientation towards the right eye as a response variable in a mixed model (Table 1A) to test for self-inspection in relation to the marking treatment. Cbind was used to create a binary response variable by combining the duration when presenting the right side of the head to the mirror and the total duration of the interaction. Dolphin identity, facility, and marking treatment were incorporated as fixed effects but a model with only marking treatment as a fixed factor showed the best model fit using the Akaike information criterion (AIC) and was significant against the null model (Chi-square, *p* = 0.003). Repeated measurements during each test session were accounted for using session and ID as nested random effects. The diagnostic plots were checked and looked satisfactory. The same mixed model approach was used to explore the side orientation towards the reflective water surface in relation to the marking treatment. To account for repeated measurements within an individual, session number was incorporated as a random effect. The selected model (using AIC) included treatment as a fixed factor (Chi-square, *p* = 0.002). The mean durations of mirror interactions between different marking treatments were compared using Mann–Whitney *U* tests in IBM SPSS Statistics 23.

## Results

**Fig. 3:**
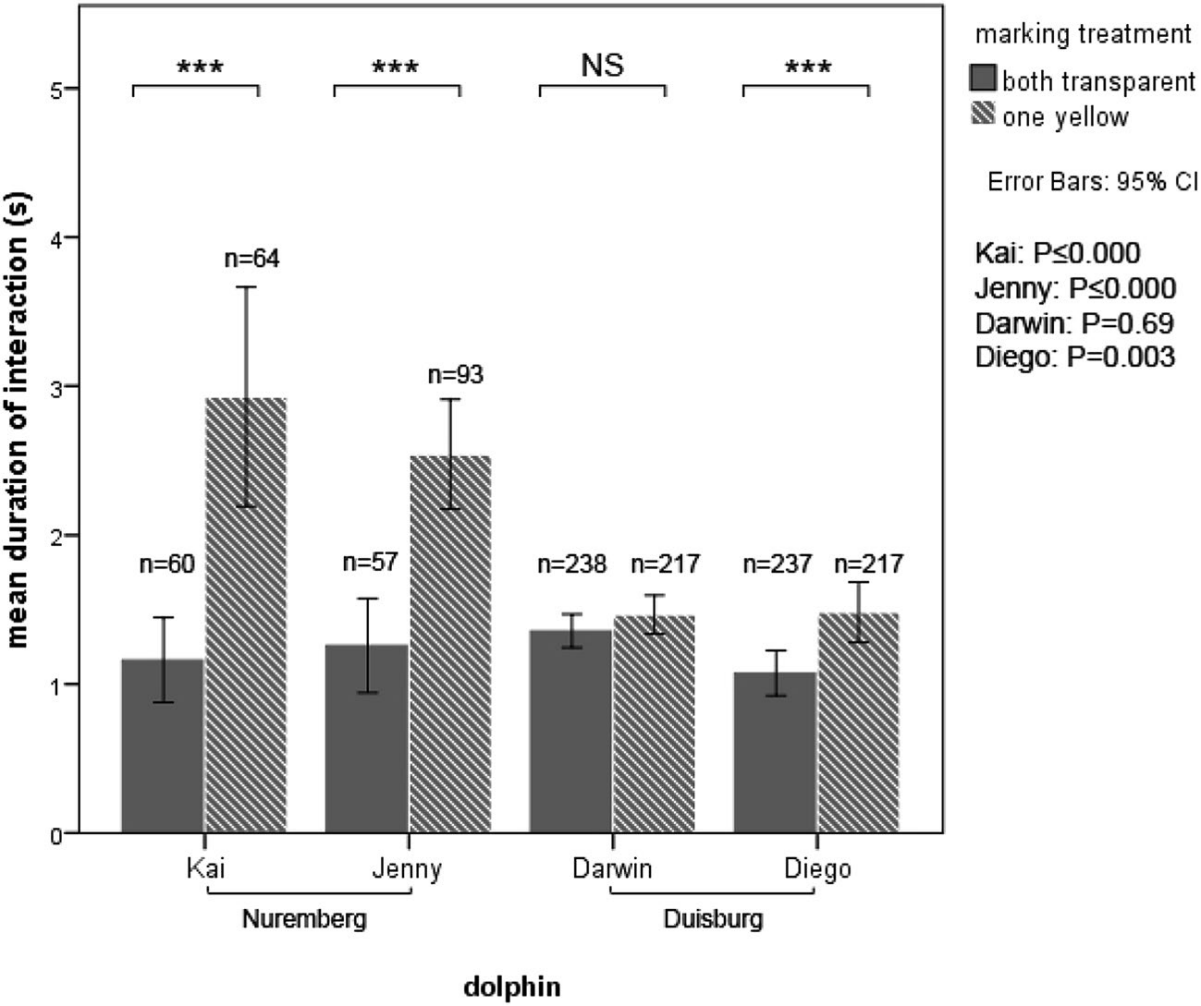
Effects of marking treatment on the duration of interaction with the mirror. Mean duration of interaction with the mirror reflection after receiving two transparent (solid bars) or one transparent and one yellow marking (hashed bars). Mann–Whitney *U* test: ***p ≤ 0.001, NS = non-significant. *N* = number of looks at the mirror

**Fig. 4:**
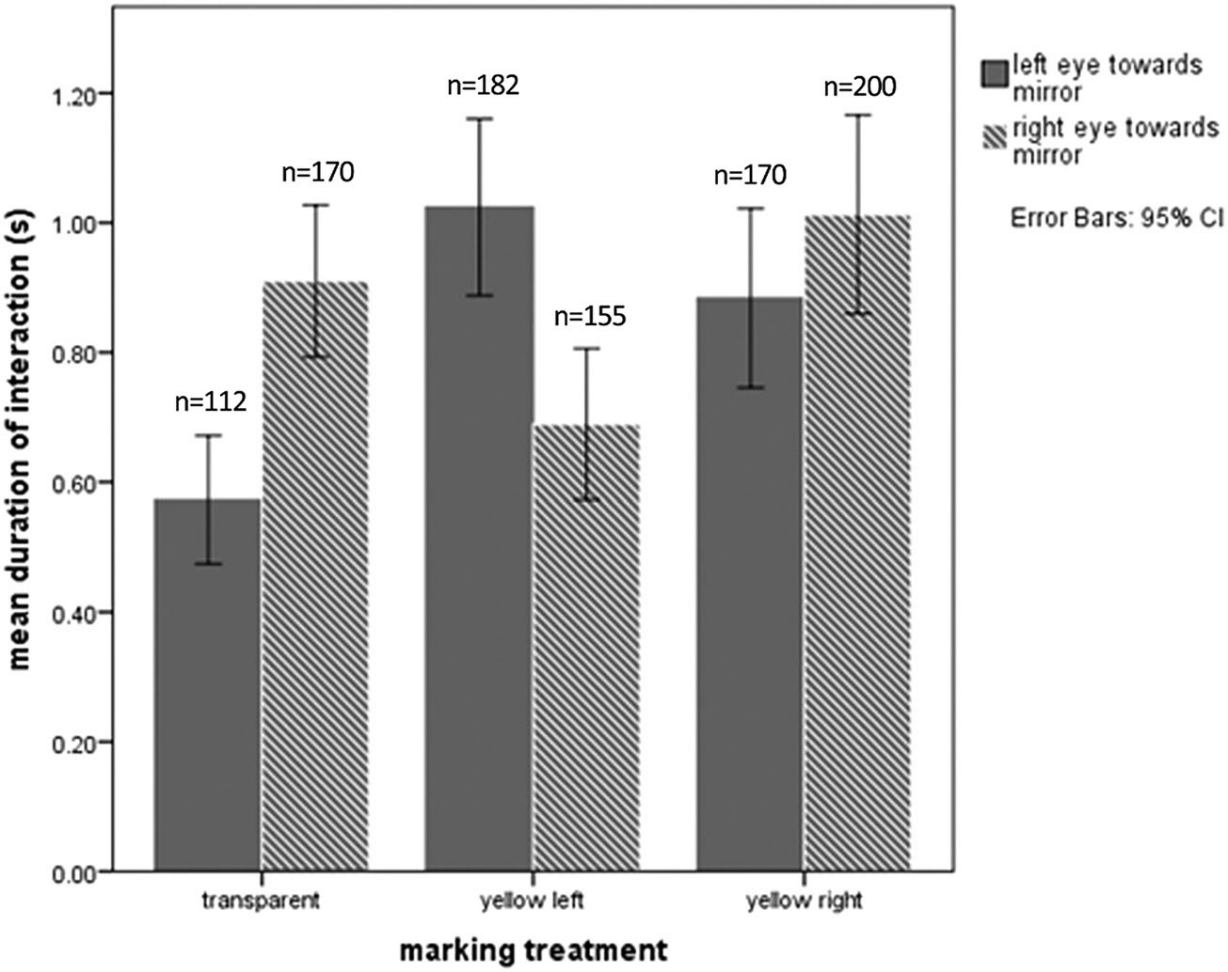
Looking time in different experimental conditions. The results show a right preference due to the preferred swimming direction of the animals, because passes without stopping at the mirror were still counted as a look at the mirror (see “Methods”). Statistical significance of different conditions is given in Table 1

All dolphins mostly had brief interactions with the mirror (mean duration per inspection: 1.48 s, ± 0.04 SE, max. 13 s). Despite these short inspection times, three out of the four dolphins spent significantly more time in front of the mirror when marked with yellow dye on one side than when marked with transparent dye on both sides (MW *U* tests for each individual, *p* < 0.001 for 3 out of 4 animals) (Fig. 3). The mixed model showed a significant effect of the left and transparent marking treatment on side orientation towards the mirror (Table 1A). A preference for counter-clockwise circling of the three males resulted in a high exposure of the right eye to the mirror even in the “transparent” condition but this was inverted when the left eye was marked yellow, suggesting that the marked eye was inspected selectively (Fig. 4). There was also an overall increase in right eye inspection time with the right eye marked, but due to the right preference this was not significant. The two animals with access to the mirror backside interacted significantly more with the reflective side of the mirror (Wilcoxon signed-rank test: *n* = 31, *Z* = − 3.785, *p* ≤ 0.000).

**Table 1:** Summary of generalised linear mixed effects models for side orientation towards (A) the mirror and (B) the reflective water surface. Scale of the response variable was used to present the model coefficients (binomial distribution and logit link function)

	Marking treatment	Coefficient *e*^*β*^	CI		*p*
			2.5%	97.5	
A) Mirror interaction (ALL)	Transparent	0.74	0.66	0.82	< 0.000
	Left	0.78	0.66	0.90	< 0.001
	Right	0.92	0.79	1.06	NS
B) Surface interaction (Darwin)	Transparent	0.80	0.37	1.76	NS
	Left	0.55	0.41	0.73	< 0.000
	Right	1.71	1.26	2.31	< 0.000

**Fig. 5:**
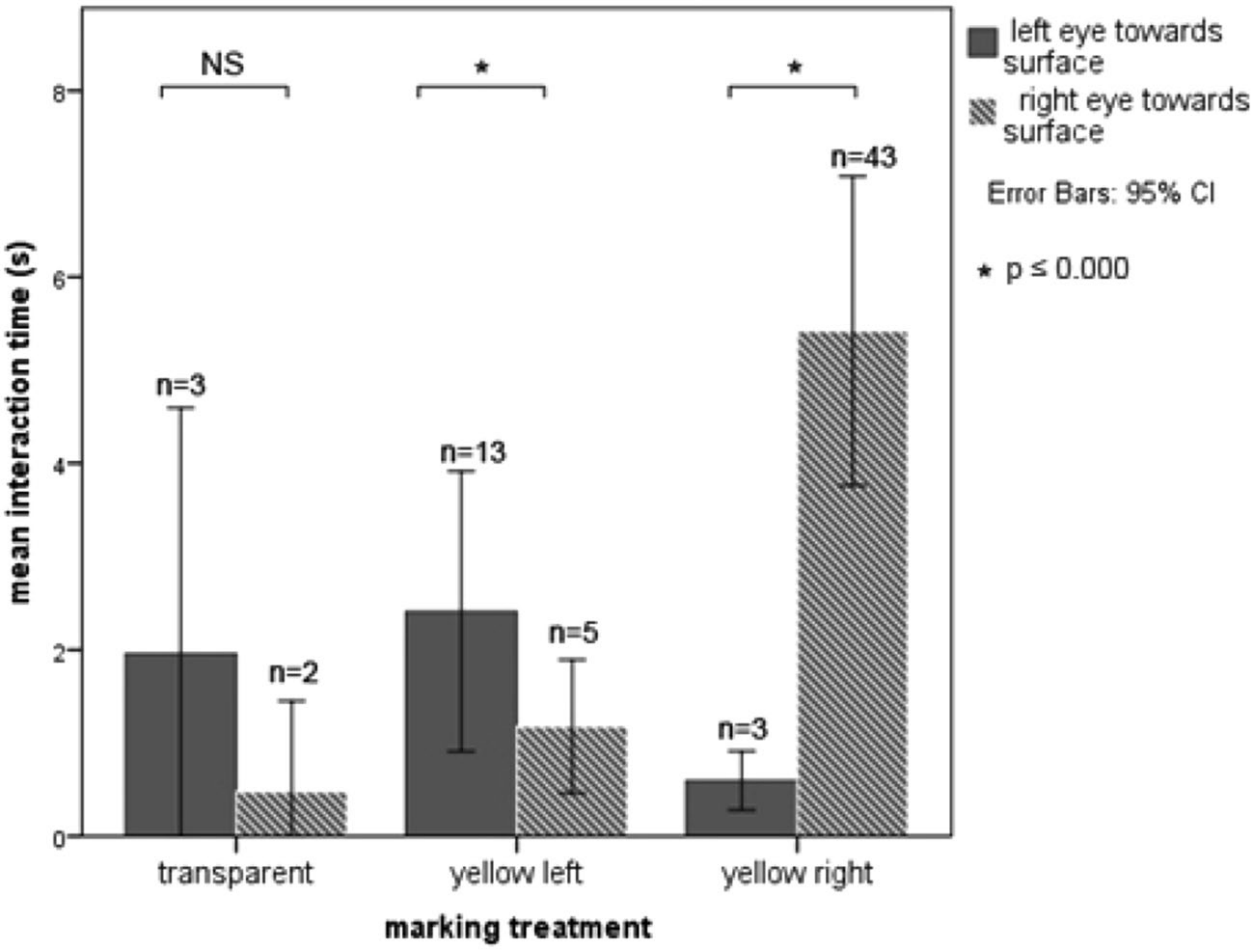
Looking times at the water surface for one animal that did not use the mirror to inspect its marks. The animal clearly turned the marked eye more towards the water surface than the sham-marked eye. Significance levels come from GLMM results

Since the water surface is a good reflector and likely gave all dolphins extensive experience with mirrors before any tests, we also timed all cases in which an animal turned one or the other eye towards the water surface during the experiment. The one individual (Darwin) that did not use the mirror repeatedly positioned itself within 1 m of depth and turned from side to side during marking trials. These interactions with the surface occurred more frequently and were significantly longer in duration when the individual had a yellow marking versus only transparent markings (Fig. 5). When marked around the left eye Darwin spent more time looking at his left eye and turned significantly less often to the right side, and when marked right he turned significantly more often towards the right side versus his left (Table 1B). Transparent markings had no significant effect on the side orientation (Fig. 5). One of the other dolphins showed the same behaviour but in a corner that was not easily observable so that we could not measure the time spent in this activity. The remaining two dolphins were the ones at Tiergarten Nürnberg and were not observed to show this behaviour near the water surface. However, not all parts of the pool for these two animals were covered by our cameras, so that we cannot be certain it did not occur.

In both facilities, the individuals were able to observe each other’s marks and behaviour in front of the mirror through meshed underwater gates, but they could not see their own or the experimental dolphin’s reflection in the mirror other than in their own test session. In Nürnberg, the individual that was not tested was not observed to be spending time close to the underwater gate. In Duisburg, this did occur close to the separating net and we used these situations as a control to investigate whether the animals were merely interested in seeing dolphins with yellow marks rather than reacting to their own specific reflection in the mirror. We found no significant difference in the duration of looking at the other dolphin between the different treatments (MW *U* tests, n.s.). Thus, neither dolphin looked at the marked animal more if it was marked with yellow dye compared to transparent dye. The separator in Duisburg was a thin rope net across the entire pool, so that animals could see each other well. Swimming direction did not matter here since the mark was visible from across the pool.

## Discussion

Our results confirm that dolphins react to marks on their bodies when seeing themselves in a mirror. We introduced a side control in which the animal had to make a clear decision to turn one way or another depending on the marking condition. This is as close as we can come to the active choice other species make when touching the mark on their bodies. The focus on looking behaviour also required a test to investigate the attractiveness of marks to dolphins in general. In finding that dolphins looked preferentially at yellow marks on themselves in a reflective surface rather than on other animals, we provide clear evidence that dolphins are capable of passing this modified mirror-mark test.

Our data provide several contrasts to findings in previous mirror-mark studies. Most notably the animals did not spend large amounts of time in front of the mirror and did not exhibit what is often called contingency behaviour. The reported long durations of mirror interactions and the absence of habituation to the mark in dolphins (Morrison and Reiss 2018; Reiss and Marino 2001) has been seen as odd in response to a non-consequential mark (Gallup and Anderson 2020). Our results meet the expectation that animals would lose interest in such a mark quickly. To measure looking duration, we had to create thresholds to define when we thought the animal could actually see its mirror image. We decided on relatively strict criteria (the dolphin eye had to have an angle of 90° ± 30° to the mirror) which could be another reason for shorter exposure times measured here than in previous studies. Contingency behaviour consists of repetitive sequences of behaviour that scientists have interpreted as evidence that the animal is testing the coherence between its own movements and those of the mirror image (but see Gallup and Anderson 2020). Another explanation is that the animal is caught in a feedback loop when trying to match the image in the mirror which leads to oscillatory repetition of behaviour. A more general point is previous experience with mirrors. Since both our test facilities had indoor areas in which underwater windows act as reflectors and the water surface can act as a mirror, our animals had extensive experience with their reflections in everyday life. We think that short looking times at the mirror and the lack of contingency behaviour were indicators that mirrors were not novel to them.

In our tests, we only used fully opaque rather than one-way mirrors. However, in a pilot study, we did present one-way mirrors at underwater windows. In these pilot tests, the occurrence of behaviour that is labelled self-directed in other studies appeared to be associated with using one-way mirrors. Slight differences in brightness between the water body and the room behind the mirror as well as a close approach allowed the animal to see through the reflective side into the room behind. We found that the uncontrolled influences from the backside of a one-way mirror and a general interest in known people around a pool often found in dolphins make one-way mirrors with people behind them unsuitable for testing dolphins for mirror self-recognition.

For the first time, we found evidence that mirrors may be used by dolphins in their natural environment. One of the animals appeared to spontaneously use the water surface as a mirror and showed a significant orientation towards the marked side while stationed very close to the water surface. The dolphin showed this behaviour exclusively within marking sessions and never during habituation sessions when no mark was applied. Just as above the water surface, light is reflected at the boundary between water and air, creating a mirror image below the water surface (Wolf and Krötzsch 1995). The degree to which the surface can function as a mirror for an animal is influenced by underwater visibility (range in which the reflection can be seen), the stillness of the surface, and light conditions inside and outside of the water. This is true both for the captive as well as the natural environment of dolphins, so that all dolphins have access to reflecting surfaces and potential experience with their own mirrored image. However, when looking upwards to the water surface, dolphins will likely see a clear cone of light with a width of about 98° while everything around this manhole will be reflected like in a mirror (Dibble et al. (2017). Thus, dolphins will not be able to see their head while approaching a flat air–water interface, but could undoubtedly see reflections of their posterior body as well as other dolphins that swim along. However, even minor ripples or waves can locally distort the image and could create transient views of the area around their eyes. That this looking-up behaviour only occurred in mark sessions and only with the marked eye directed at the surface is intriguing but requires further study to clarify what the animal could see. It is uncertain whether dolphins in the wild use the water surface to inspect themselves (e.g. when remoras attach themselves to a dolphin). Future studies should look at the potential use of the water surface in this way. If this is a common behaviour, dolphins may have a unique pre-disposition to use mirrors that could influence their perception of themselves.

Our demonstration of mark-directed inspection behaviour leaves the question of what it tells us about self-recognition. Since its initial introduction by Gallup (1970), the mark-and-mirror test has been seen as a highly important innovation in comparative psychology, but in parallel also witnessed various controversies. We see four areas of dispute that are relevant here. The first question concerns the functional interpretation of this test. Often it is seen as an indicator of self-awareness/consciousness (Murray et al. 2022; Mashour and Alkire 2013). Meanwhile, compelling behavioral, imaging-based, and electrophysiological markers for the presence of conscious perception in humans have been established (e.g. Mashour et al. 2020). Using the identical experimental procedures, macaques produce the same behavioural and neuroscientific indicators of conscious perception as humans (Dehaene et al. 2017; van Vugt et al. 2018). Recently, the same behavioural and electrophysiological markers were also demonstrated in carrion crows (Nieder et al. 2020; Güntürkün 2021). However, passing the mark test in macaques is contested (Anderson and Gallup 2011; Chang et al. 2015), while carrion crows fail this test (Brecht et al. 2020). Thus, the consciousness interpretation of the mirror test in these two species is in contradiction with result patterns that test the same trait with a different paradigm. The same is also true when seeing the mark- and mirror test as an indicator for the ability to socially cognise. Although most children from so-called western countries pass the mark test when being 24 months or older, only few children from rural areas in Kenya do so (Broesch et al. 2011). Since neither consciousness nor social cognition is lacking in these children, the mark test seems to produce problems when cultural norms conflict with the behaviour that these children have to produce to pass the test. Thus, contextual factors can produce false negatives in the mark-and-mirror test when applied to children.

Our second question concerns potentially high false-negative rates of the test in non-human animals. In all mark test studies with different animal species, only a (small) subset of individuals passes the test (Povinelli et al. 1993, 1997; Prior et al. 2008), while initially successful individuals do not necessarily pass during test repetitions (Plotnik et al. 2006). These and many more studies indicate that the mark test seems to produce a substantial amount of falsely negative results. As also outlined by De Veer and van den Bos (1999) it is unclear whether a failure in species and individuals is due to methodological circumstances or is clearly related to an absence of a cognitive ability. Our study showing a clearly detectable but seemingly subtle difference in looking time might pave the way to look at more subtle indicators in other species that are clearly measurable and therefore less prone to misinterpretation.

The third question concerns the association between one’s own body and its mirror image. Usually, the dictum of the mark test is that no formal learning procedure is allowed and relevant associations have to be acquired during first exposures to a mirror. In macaques, however, a short training with an irritant laser that can be seen in a mirror seems to create a step-function of their subsequent spontaneous behaviour (Chang et al. 2015). After this training, these macaques make spontaneous use of mirrors to inspect parts of their body that they normally could not observe directly (Chang et al. 2015). Thus, this rather simple training enables these animals to show a spontaneous behaviour akin to chimpanzees (Gallup 1970). It is important to note that the study of Chang et al. (2015) is different from that of Epstein et al. (1981). In the latter, pigeons were conditioned over a lengthy period of time to peck a mark on their body that they could not see without a mirror. After successful training, these pigeons made no spontaneous use of mirrors: they simply had learned an S-R association but had not acquired anything that is remotely relevant for the mark test.

The fourth question is whether self-recognition is a binary trait or a continuous one along evolutionary gradients (de Waal 2019). Capuchin and spider monkeys (de Waal et al. 2005; Murray et al. 2020) as well as pigeons (Wittek et al. 2021) fail the mark test but do not show social behaviour towards their mirror image. Instead, they treat it as an uncanny individual rather than as a stranger. These results indicate that the binary fail-or-pass outcomes of the mark test do not necessarily capture the evolutionary much more likely graded result pattern which would ascribe different levels of mirror-understandings to different species that live in different ecological contexts (Clary and Kelly 2016; Kohda et al. 2022). Obviously, different scientists might interpret this and related points differently.

To summarise, we are confident that the mark test is a highly useful test for self-recognition in non-human animals when positive data are obtained. In our study, we obtained positive results for dolphins. Not every animal is necessarily motivated to remove marks on itself (Kakrada and Colombo 2022) in which case looking times might be more informative than other actions even in animals that can reach the mark. Furthermore, the recognition of oneself in a mirror does not necessarily imply full self-awareness or consciousness (Kohda et al. 2019). Kinaesthetic matching (Mitchell 1993) or the ability to collate different representations (Suddendorf and Butler 2013) have not been sufficiently ruled out as contributing to the behaviour that is observed. The form of self-recognition demonstrated in our study may be context-specific and does not necessarily imply a universal concept of self, but even if we accept more basic explanations, it still requires the dolphin to have mental representations of its own movements or body that the animal can compare across modalities with the perceived body of another dolphin (the one seen in the mirror) (Burge 2013). This distinction between the animal’s own body and that of another constitutes an important step in the evolution of self-awareness.

## Data Availability

The datasets generated during the current study are available from the corresponding author on reasonable request.
